# Cardiac safety of indacaterol in healthy subjects: a randomized, multidose, placebo- and positive-controlled, parallel-group thorough QT study

**DOI:** 10.1186/1471-2466-11-31

**Published:** 2011-05-26

**Authors:** Sanjeev Khindri, Ronald Sabo, Stuart Harris, Ralph Woessner, Simon Jennings, Anton F Drollmann

**Affiliations:** 1 Novartis Horsham Research Centre, Horsham, West Sussex RH12 5AB, UK; 2 Novartis Pharmaceuticals Corporation, East Hanover, NJ 07936, USA; 3 SeaView Research Inc, Miami, FL 33126, USA; 4 Novartis Institute of Biomedical Research, Basel, Switzerland

## Abstract

**Background:**

Indacaterol is a novel once-daily ultra long-acting β_2_-agonist for the treatment of chronic obstructive pulmonary disease. It is known that β_2_-agonists, like other adrenergic compounds, can prolong the QT-interval. This thorough QT/QTc study (as per ICH E14 guideline) evaluated the effect of indacaterol on the QT interval in healthy subjects.

**Methods:**

In this randomized, double-blind, parallel-group, placebo- and positive-controlled (open-label moxifloxacin) study, non-smoking healthy subjects (18-55 years, body mass index: 18.5-32.0 kg/m^2^) were randomized (4:4:2:4:1) to 14-day treatment with once-daily indacaterol (150 μg, 300 μg, or 600 μg), placebo, or placebo/moxifloxacin (double-blind 14-day treatment with placebo and a single open-label dose of 400 mg moxifloxacin on Day 14). The primary endpoint was the change from baseline on Day 14 in QTcF (QT interval corrected for heart rate using Fridericia's formula).

**Results:**

In total, 404 subjects were randomized to receive indacaterol (150 [n = 108], 300 [n = 108], 600 μg [n = 54]), placebo (n = 107), or placebo/moxifloxacin (n = 27); 388 subjects completed the study. Maximal time-matched mean (90% confidence intervals) treatment differences from placebo in QTcF change from baseline on Day 14 were 2.66 (0.55, 4.77), 2.98 (1.02, 4.93) and 3.34 (0.86, 5.82) ms for indacaterol 150 μg, 300 μg and 600 μg, respectively. Study sensitivity was confirmed with moxifloxacin demonstrating a significant maximal time-matched QTcF prolongation of 13.90 (10.58, 17.22) ms compared to placebo. All indacaterol doses were well tolerated.

**Conclusion:**

Indacaterol, at doses up to 600 μg once daily (2-4 times the therapeutic dose) does not have any clinically relevant effect on the QT interval.

**Trial Registration:**

ClinicalTrials.gov: NCT01263808

## Background

Indacaterol is a novel, inhaled, ultra long-acting β_2_-agonist (LABA) [1] that is approved in a number of countries, including throughout the European Union (EU), at doses of 150 and 300 μg once-daily (OD) for the maintenance bronchodilator treatment of airflow obstruction in adult patients with chronic obstructive pulmonary disease (COPD). It is a partial agonist at the human β_2_-adrenoceptor with a binding affinity similar to that of formoterol, and an intrinsic activity higher than that of salbutamol and salmeterol [2,3]. The efficacy and safety of indacaterol in patients with COPD have been previously evaluated in number of studies, where once-daily indacaterol has demonstrated 24-h bronchodilation with a rapid onset of action [4-8].

The current International Conference on Harmonisation (ICH) of Technical Requirements for Registration of Pharmaceuticals for Human Use E14 Guidance [9] recommends that all new non-antiarrhythmic drugs that are systemically bioavailable should undergo a rigorous electrocardiographic evaluation as part of a controlled clinical study (that is a "thorough QT study"), to evaluate their potential QT liability. An abnormal QT-interval morphology is considered to be a surrogate biomarker for the proarrhythmic risk of non-antiarrhythmic drugs. Results from a meta-analysis of 33 randomized placebo-controlled trials (N = 6623) evaluating the arrhythmogenic potential of β-agonists [10] suggest that inhaled β_2_-agonists have a potential to increase heart rate and the incidence of ventricular arrhythmias in patients with obstructive airway disease. It is, therefore, especially important to conduct a thorough QT study as part of the development of an inhaled β_2_-agonist.

A thorough QT study is typically carried out in healthy volunteers as opposed to individuals at increased risk of arrhythmias. It is driven by a test of non-inferiority on the rate-corrected QT interval (the QTc interval) and is considered negative when the largest time-matched mean difference in the QTc interval between the study drug and placebo is less than or equal to (approximately) 5 ms, with the upper bound of the 95% one-sided confidence interval (CI) excluding 10 ms [9].

The present study, which, to the best of our knowledge, is the largest thorough QT study conducted thus far with any β_2_-agonist, was designed primarily to characterize the maximum mean QTc interval prolongation following treatment with three different doses of indacaterol in healthy individuals. Safety and tolerability and pharmacokinetics of indacaterol were also assessed.

## Methods

This was a randomized, multiple-dose, placebo- and positive-controlled, parallel-group study conducted at a single clinical research center in the US. The study design was in compliance with the recommendation of the ICH E14 guideline [9] for thorough QT studies. The study was approved by the institutional review board (Independent Investigational Review Board; Sea View Research, Inc.) of the participating study center and was conducted in accordance with the ethical principles embodied in the Declaration of Helsinki (1989).

### Subjects

The study population consisted of healthy, nonsmoking male and female subjects aged between 18 and 55 years (inclusive), with a body mass index within the range of 18.5-32.0 kg/m^2 ^and weight ≥50 kg at screening. Individuals with a history of or current ECG abnormalities (PR >240 ms; QRS complex >110 ms; QTc interval [Fridericia's; QTcF] >430 ms for males and >440 ms for females; or any significant morphological changes other than nonspecific T-wave changes) were excluded from the study. In addition, those with serum potassium <3.5 mmol/L or magnesium <0.8 mmol/L; recent blood donation or blood loss; or a history of diabetes mellitus, hyperthyroidism, impaired renal function, drug or alcohol abuse, or any significant illness within 2 weeks of dosing were also excluded. All subjects provided written informed consent prior to taking part in the study.

### Study design and treatments

The study comprised a screening period (up to 21 days), a baseline visit (Day -1), a 14-day randomized treatment period (Days 1-14), and an end-of-study evaluation at 7 days after the last dose. At the start of the randomized treatment period, eligible subjects were randomized to one of the following five treatment groups in a 4:4:2:4:1 ratio: indacaterol 150 and 300 μg (therapeutic doses), indacaterol 600 μg (supratherapeutic dose), placebo, and placebo/moxifloxacin. Moxifloxacin, a fluoroquinolone antimicrobial agent, was used as a positive calibrator to determine the assay sensitivity in this study. This dose of 400 mg represents the daily therapeutic dose of moxifloxacin, single doses of which have been consistently associated with an increase in mean QT interval vs placebo in excess of 5 ms in healthy volunteers [11].

Indacaterol and matching placebo were administered once daily for 14 days via a single-dose dry powder inhaler (SDDPI) under double-blind conditions. Subjects randomized to the placebo/moxifloxacin arm received double-blind placebo via SDDPI for 14 days, with a single open-label oral dose of moxifloxacin 400 mg on Day 14.

### Concomitant treatment

Except for paracetamol and any medication needed to treat adverse events (AEs), no medication other than study drug was allowed from the start of screening until the completion of all evaluations in the study.

### Assessments

#### ECG profiling

Serial 12-lead surface ECGs were obtained using digital ECG equipment (ELI 250™, Mortara Instrument Inc., Milwaukee, WI, USA) at baseline (Day -1) at the equivalent of predose and 1 and 12 h post-dose. During the treatment period, three ECG recordings were collected at each of the following times on Days 1 and 14: at predose, and at 10, 20, and 40 min, and 1, 2, 3, 4, 6, 12, and 24 h post-dose. All ECGs were recorded after subjects had been resting in the supine position for at least 15 min (or 8 min for the 10 min post-dose reading). The digital ECG recordings were transmitted electronically to a central facility for blinded manual interpretation and analysis. All ECG readers were blinded to all elements of the study, including treatment assignments and subject demographics.

#### Pharmacokinetic assessments

Pharmacokinetic samples were taken to determine the relationship between indacaterol concentration and QTc interval. Serial blood samples were collected on Days 1 and 14 at predose and at 10, 20, and 40 min, and 1, 2, 3, 4, 6, 12, and 24 h post-dose. Serum was obtained from all blood samples by centrifugation and kept frozen at ≤ -20°C until analysis. Serum indacaterol was measured using a liquid chromatography/mass spectrometry/mass spectrometry method; the lower limit of quantification for indacaterol was 10 pg/mL. The maximum (peak) serum concentration (C_max_) and the area under the concentration-time curve from 0 to 24 h post-dose (AUC_0-24h_) were determined on Days 1 and 14, and were presented as the mean and standard deviation (SD).

#### Safety assessments

Safety assessments included recording of AEs and serious adverse events (SAEs) with severity and relationship to study drug; collection of clinical laboratory data for urinalysis, hematology, and blood chemistry (including serum potassium and plasma glucose); and regular assessments of vital signs, ECGs, and body weight.

#### Sample size calculation and statistical analysis

The primary endpoint for the study was the QTcF interval (QT interval corrected for heart rate using Fridericia's formula, QT/RR^0.33^). This formula is recommended for investigating the effect of drugs that may affect pulse rate [9,12,13]. For each of three ECGs taken at each nominal time, the QT and RR intervals were measured from three consecutive QRST complexes from lead II. The mean of the triplicate ECG value was then calculated.

The primary objective of this study was to compare indacaterol 150, 300 and 600 μg with placebo in terms of the change in QTcF interval from baseline at each of the 10 post-dose time points on Day 14. Baseline QTcF interval was defined as the average of the pre-treatment measurements taken on Day -1 and the predose measurement on Day 1.

The sample size calculation was based on a residual standard deviation of 10.95 ms for the primary endpoint observed in a previous indacaterol study (data on file). Assuming a 5 ms difference between indacaterol 150 μg or 300 μg and placebo in terms of change from baseline in QTcF interval at the first four post-dose assessment time points on Day 14 and no difference at the next six time points, it was calculated that a sample size of 102 subjects was required in each treatment group to demonstrate that all upper one-sided 95% confidence intervals were below 10 ms.

For the comparison of indacaterol 600 μg versus placebo, 52 subjects in each treatment arm were required in order to show that all upper one-sided 95% confidence intervals were <20 ms. This calculation assumed an estimated difference of 13 ms between indacaterol 600 μg and placebo in terms of change from baseline in QTcF interval at the first four assessment time points and no difference between the treatments at the next six time points.

For the comparison between moxifloxacin and placebo, 26 subjects per treatment arm were required to conclude an assay sensitivity with 95% power based on the assumption that the difference between moxifloxacin and placebo for QTcF interval change from baseline was at least 5 ms and that this difference could be detected at least at 4 of the 10 post-dose assessment time points on Day 14. Assuming a dropout rate of approximately 10%, the number of subjects to be randomized was 116, 116, 58, 116, and 29, for indacaterol 150, 300, 600 μg, placebo, and placebo/moxifloxacin, respectively.

The absence of any meaningful effect of indacaterol 150 and 300 μg on cardiac repolarization was to be concluded if the upper limit of all the 90% two-sided CIs (equivalent to the upper limit of a 95% one-sided CI) for the mean difference in the change in QTcF interval from baseline between indacaterol (150 and 300 μg) and placebo at each time point was <10 ms. A similar analysis was performed for the supratherapeutic dose of indacaterol (600 μg) with the noninferiority margin set at a prespecified constant of 20 ms for the upper limit of the 90% two-sided CIs. Confidence intervals were calculated from pairwise determination of the pooled standard error and the 10^th ^and 90^th ^percentiles of the t-distribution with appropriate degrees of freedom.

The average QTcF intervals (average of all scheduled post-dose QTcF measurements up to 24 h post-dose) and the peak QTcF intervals (highest QTcF interval value observed over the period of 10 min to 24 h post-dose) were calculated for all treatment groups on both Days 1 and 14. In addition, the incidences of absolute QTcF interval values >450, >480, and >500 ms were summarized for each treatment group, as were the incidences of changes from baseline in QTcF interval >30 and >60 ms.

#### Concentration-effect modeling

To determine the relationship between steady-state serum concentration of indacaterol and QTcF interval, change from baseline in QTcF interval was plotted against the corresponding indacaterol concentration for each time point on Day 14 (including all observations under placebo and all indacaterol doses). A linear relationship between the two was established by plotting a regression line, with the upper 95% simultaneous confidence band also presented.

## Results

### Subject disposition, demographics, and baseline characteristics

The study was conducted between April (first subject enrolled) and August 2008 (last subject completed). A total of 404 subjects were randomized, receiving indacaterol 150 μg (n = 108), indacaterol 300 μg (n = 108), indacaterol 600 μg (n = 54), placebo (n = 107), or placebo/moxifloxacin (n = 27); 388 subjects (96%) completed the study (94%, 94%, 98%, 97% and 100% for indacaterol 150, 300 and 600 μg, placebo and placebo/moxifloxacin, respectively). The most common reason for premature discontinuation was loss to follow up (n = 6) followed by withdrawal of consent (n = 4), AEs (n = 3), protocol deviations (n = 2), and abnormal test results (n = 1).

The treatment groups were well-balanced in terms of demographic and baseline characteristics, except that the proportion of Caucasian subjects (96%) was higher in the placebo/moxifloxacin group as compared with the other four treatment groups (85-89%) (Table 1).

**Table 1:** Demographic and baseline characteristics of the study participants

Demographic parameter	Indacaterol			Placebo	Placebo/moxifloxacin
			
	150 μg	300 μg	600 μg		
	**N = 108**	**N = 108**	**N = 54**	**N = 107**	**N = 27**
Male, n (%)	76 (70.4)	75 (69.4)	37 (68.5)	76 (71.0)	18 (66.7)
Female, n (%)	32 (29.6)	33 (30.6)	17 (31.5)	31 (29.0)	9 (33.3)
Age (yrs), mean (SD)	37.4 (9.98)	39.0 (10.28)	39.1 (9.65)	38.5 (10.46)	39.0 (10.58)
Body mass index (kg/m^2^), mean (SD)	26.6 (2.85)	26.9 (2.78)	26.7 (2.43)	26.8 (2.66)	26.0 (3.17)
Weight (kg), mean (SD)	76.6 (11.97)	77.4 (11.50)	76.4 (10.26)	78.0 (11.64)	73.2 (13.50)
Race					
○Caucasian (%)	96 (88.9)	95 (88.0)	46 (85.2)	95 (88.8)	26 (96.3)
○Black (%)	11 (10.2)	13 (12.0)	8 (14.8)	12 (11.2)	1 (3.7)
○Other (%)	1 (0.9)	0	0	0	0

### QT interval data

#### Effect of indacaterol and moxifloxacin on QTcF interval

**Figure 1:**
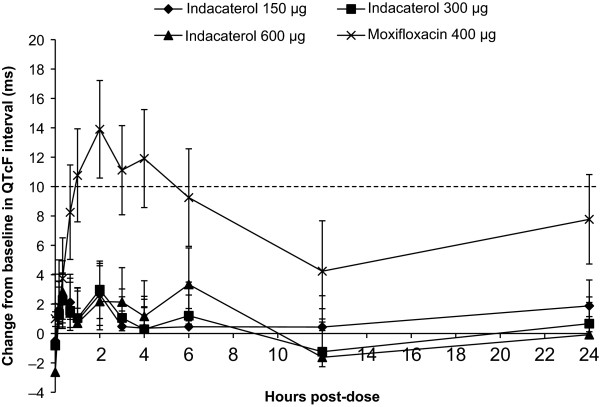
**Difference from placebo in mean change from baseline in QTcF on Day 14**. Data presented are mean and 90% confidence interval. Dotted horizontal line corresponds to the 10 ms threshold.

The mean differences in time-matched changes from (time-averaged) baseline in QTcF interval between all indacaterol doses and placebo on Day 14 were well below 5 ms (the threshold of regulatory concern) at each time point (Table 2, Figure 1), with no discernible dose-relationship. The maximal time-matched mean differences from placebo in QTcF change from baseline for indacaterol 150 μg (2.66 ms) and 300 μg (2.98 ms) doses were observed at 2 h post-dose (Table 2). The corresponding maximal difference between indacaterol 600 μg and placebo (3.34 ms) was observed at 6 h post-dose (Table 2). For all three indacaterol doses, the corresponding upper bounds of the two-sided 90% CIs (equivalent to the upper bound of a one-sided 95% CI) excluded 10 ms indicating a negative thorough QT study.

**Table 2:** Difference versus placebo in the change in QTcF interval from baseline on Day 14

Time post- dose	Indacaterol			Placebo/moxifloxacin vs placebo
		
	150 μg vs placebo	300 μg vs placebo	600 μg vs placebo	
	**Mean (90% confidence intervals), in ms**			

10 min	1.62 (-0.34, 3.57)	1.26 (-0.64, 3.16)	1.56 (-0.83, 3.94)	1.84 (-1.32, 5.01)
20 min	2.23 (0.34, 4.12)	2.24 (0.45, 4.03)	2.84 (0.72, 4.95)	3.74 (0.97, 6.51)
40 min	2.12 (0.21, 4.03)	1.54 (-0.41, 3.49)	1.40 (-0.96, 3.76)	8.25 (5.04, 11.47)
1 h	0.89 (-1.12, 2.89)	1.05 (-0.99, 3.09)	0.70 (-1.72, 3.12)	10.76 (7.60, 13.93)
2 h	2.66 (0.55, 4.77)	2.98 (1.02, 4.93)	2.18 (-0.25, 4.61)	13.90 (10.58, 17.22)
3 h	0.48 (-1.53, 2.50)	1.06 (-0.91, 3.03)	2.14 (-0.19, 4.48)	11.12 (8.08, 14.15)
4 h	0.36 (-1.82, 2.53)	0.29 (-1.76, 2.33)	1.17 (-1.26, 3.59)	11.91 (8.57, 15.24)
6 h	0.46 (-1.70, 2.62)	1.21 (-0.64, 3.05)	3.34 (0.86, 5.82)	9.25 (5.93, 12.57)
12 h	0.44 (-1.69, 2.57)	-1.24 (-3.26, 0.79)	-1.62 (-4.23, 0.98)	4.24 (0.82, 7.67)
24 h	1.88 (0.12, 3.64)	0.67 (-1.15, 2.49)	-0.08 (-2.27, 2.11)	7.77 (4.73, 10.82)

Moxifloxacin administration resulted in a clinically significant increase in QTcF, thereby establishing the assay sensitivity of the trial. The largest mean difference between placebo/moxifloxacin and placebo alone (13.90 ms) was observed at 2 h post-dose; the corresponding upper bound of the two-sided 90% CI (17.22 ms) exceeded the regulatory threshold at this timepoint and at several other timepoints (Table 2, Figure 1) [9].

Table 3 shows the treatment comparisons between the indacaterol doses and placebo with respect to average and peak QTcF on Days 1 and 14. As with the primary analysis, all mean values were below 5 ms, with the upper bound of all 90% confidence intervals being below 10 ms.

**Table 3:** Difference between indacaterol and placebo for average and peak QTcF on Days 1 and 14

	**Indacaterol 150 μg vs placebo**		**Indacaterol 300 μg vs placebo**		**Indacaterol 600 μg vs placebo**	
	
	**Least squares mean (90% confidence intervals)**					
	
	Day 1	Day 14	Day 1	Day 14	Day 1	Day 14
	
Average QTcF (ms)	0.66	1.32	2.09	1.04	3.71	1.00
	(-0.22, 1.53)	(-0.20, 2.84)	(1.21, 2.96)	(-0.48, 2.56)	(2.64, 4.79)	(-0.85, 2.84)
Peak QTcF (ms)	-0.21	1.23	2.04	0.38	3.57	0.45
	(-1.40, 0.97)	(-0.56, 3.02)	(0.86, 3.22)	(-1.41, 2.17)	(2.12, 5.02)	(-1.72, 2.62)

Analysis of covariance including baseline QTcF as a covariate and treatment as a fixed effect

Average QTcF is the average of all scheduled post dose QTcF measurements between dosing and 24 h post-dose

Peak QTcF is the maximum QTcF of all scheduled post-dose QTcF measurements between dosing and 24 h post-dose

#### Categorical analysis

Two subjects, one each from the indacaterol 150 μg (20 min post-dose on Day 14) and 300 μg (10 min post-dose on Day 1) dose groups, demonstrated QTcF interval values exceeding 450 ms; no values exceeded 480 ms. There were no QTcF changes from baseline >60 ms. A total of ten changes >30 ms were recorded: five with indacaterol 150 μg (one subject had three values and two others had one value >30 ms each); three with placebo (three subjects with one value >30 ms each); and two with placebo/moxifloxacin (one subject with two values >30 ms).

#### Pharmacokinetics

Indacaterol was absorbed rapidly following inhalation, with a median time to reach peak serum concentrations on Days 1 and 14 of 15 min post-dose. After 14 days of once-daily dosing, indacaterol appeared to achieve steady state. At steady state (i.e., on Day 14), C_max _and AUC_0-24h _increased proportionally over the entire dose range (150-600 μg) (Table 4).

**Table 4:** C_max _and AUC_0-24h _of indacaterol after once-daily inhalation on Days 1 and 14

	Dose (μg)	C_max _(pg/mL)	AUC_0-24h _(pg·h/mL)
		mean ± SD	mean ± SD
Day 1	150	252.9 ± 120.84	1202.0 ± 553.93
	300	537.2 ± 224.23	2639.4 ± 862.08
	600	1043.8 ± 285.47	5279.1 ± 1155.28

Day 14	150	438.6 ± 196.38	3881.7 ± 1545.39
	300	858.6 ± 264.16	8137.0 ± 2388.41
	600	1656.7 ± 540.76	15084.6 ± 3428.15

#### Concentration-QTcF interval relationship

**Figure 2:**
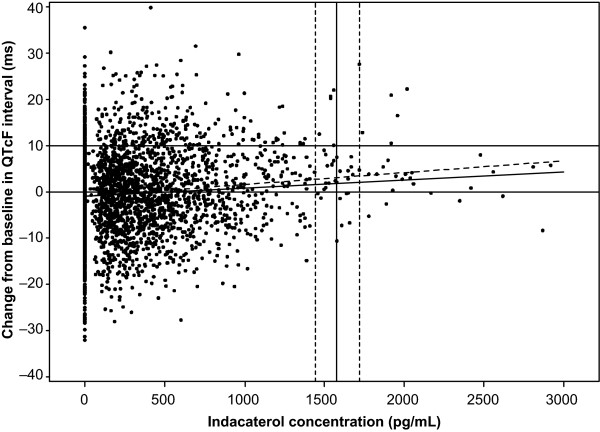
**Relationship between indacaterol concentration and QTcF change from baseline following 14-day treatment with indacaterol**. The solid diagonal line represents the estimated regression of concentration against change from baseline in QTcF interval; the dotted diagonal line represents the corresponding 95% upper confidence band. The horizontal lines show 0 and 10 ms change from baseline in QTcF interval; the vertical lines are the geometric mean and 90% confidence limits for C_max _in the indacaterol 600 μg group.

A scatter plot (Figure 2) was used to assess the relationship between Day 14 indacaterol serum concentration and change in QTcF interval from baseline following multiple dosing with indacaterol 150, 300, and 600 μg. At all concentrations up to 3000 pg/mL, the 95% confidence band of the regression line was below the line for 10 ms QTcF change from baseline. These results support the QTcF interval analysis and indicate that prolongation of QTcF interval above 10 ms is not expected at doses of indacaterol resulting in steady-state C_max _values up to 3000 pg/mL.

#### Safety results

The overall incidence of AEs was 47.2% (51/108), 54.6% (59/108), and 61.1% (33/54) with indacaterol 150 μg, 300 μg, and 600 μg, respectively, as compared with 47.7% (51/107) with placebo, and 51.9% (14/27) with placebo/moxifloxacin. The most common AE was contact dermatitis (indacaterol 150 μg: n = 31 [28.7%]; indacaterol 300 μg: n = 36 [33.3%]; indacaterol 600 μg: n = 21 [38.9%]; placebo: n = 30 [28.0%]; placebo/moxifloxacin: n = 6 [22.2%]) attributed to the repeated application and removal of adhesive ECG electrodes. AEs were mostly mild in severity and in most cases were not suspected to be study drug related. Two subjects experienced SAEs, one receiving indacaterol 300 μg (chest pain) and one receiving placebo (acute depression). Neither event was suspected to be study drug related, although both subjects were withdrawn from the study. No death was reported during the study. There were no clinically notable serum potassium or blood glucose values or any abnormalities pertaining to heart rate or other vital signs in any group.

## Discussion

In this study, indacaterol 150 μg, 300 μg, and 600 μg, administered once-daily for 14 days resulted in a maximum mean prolongation in QTcF of less than 5 ms versus placebo, with the upper limit of all two-sided 90% CIs being below 10 ms. Therefore, according to the ICH E14 guidelines [9], the present thorough QT study can be deemed negative, indicating that indacaterol has no QT effects of concern across the investigated dose range.

In terms of dose-effect relationship, the highest dose of indacaterol (600 μg) resulted in the largest QT interval prolongation on Day 14 (3.34 ms at 6 h post-dose); however, the respective 90% CIs for all indacaterol doses largely overlapped with one another suggesting the absence of any dose-QTcF relationship. The lack of dose-dependency in the investigated dose range further supported by the mean QTcF changes from baseline on Day 14, which showed similar, small (<5 ms) and transient increases in QTcF interval in each indacaterol dose group compared with placebo. Further, on Day 14 there were only marginal and non-significant changes in average and peak QTcF intervals with all three doses of indacaterol, with the concentration-QTcF interval analysis indicating that a prolongation above 10 ms is not expected at doses of indacaterol resulting in steady-state C_max _values up to 3000 pg/mL. This is particularly relevant, given that according to the prescribing information for formoterol and salmeterol, at high doses these other β_2_-agonists are associated with QT interval prolongation. Since this study did not include any other LABAs, one cannot draw any direct conclusions from the results about the relative cardiovascular safety of indacaterol, salmeterol and formoterol. Such a comparison is of interest, given that these three LABAs have different intrinsic efficacies [14]. However a 12-month study in which patients with COPD were randomized to receive indacaterol 300 μg or 600 μg, formoterol or placebo, showed that all four treatments had similar good overall safety profiles [8]. Furthermore, given that the current study recruited healthy volunteers (in accordance with ICH E14 guidelines), the earlier 12-month study is useful in that it also provides evidence of cardiovascular safety in patients with COPD and a range of comorbid conditions (including hypertension and Type II diabetes mellitus). In addition, in a recent post-hoc analysis of the indacaterol trial database, indacaterol did not increase the risk of cerebrovascular or cardiovascular adverse events compared with placebo, formoterol or salmeterol [15].

In accordance with the ICH E14 guidelines, moxifloxacin was included in the study as a positive control, and administration of a single 400 mg dose resulted in a clinically significant increase in QTcF interval, thereby establishing the assay sensitivity of the trial. QTc interval prolongation with moxifloxacin in this trial was similar to that reported previously [11].

The indacaterol doses administered in this study (150 μg, 300 μg, and 600 μg) were selected based on a dose finding study which used an adaptive seamless study design [16], and which identified the 150 and 300 μg doses for further development. These two doses were subsequently approved in a number of countries, including throughout the European Union, for maintenance treatment of COPD. Previous studies have demonstrated that patient age, weight and ethnicity have no clinically relevant effect on indacaterol pharmacokinetics (C_max _and AUC; data on file). Further, metabolic drug interactions raise the systemic exposure of indacaterol by a maximum of two-fold (data on file). In this context, the absence of prolongation of QTcF above 10 ms with the 600 μg dose (i.e., two- to four-fold higher than the approved EU doses) is a relevant finding for the safety profile of indacaterol.

Notably, in the current study no change from baseline in QTcF interval of >60 ms was observed with any of the indacaterol doses, and nor were any QTcF interval values >480 ms observed. These results also demonstrated that indacaterol did not prolong QTc interval and further suggests that there were no outliers in terms of QTc interval prolongation. Indeed in a previous study in patients with mild or moderate COPD, even at single doses as high as 3000 μg indacaterol had minimal effects on the QTc interval [17].

## Conclusion

The results of this thorough QT study carried out in over 400 healthy subjects indicate that indacaterol, at doses up to 600 μg once daily (2-4 times the therapeutic dose) does not have any clinically relevant effect on the QT interval.

## Competing interests

SH has been reimbursed by Novartis, the sponsors of this clinical trial, for attending conferences, and is the Medical Director of SeaView Research, Inc., which received a research grant from Novartis for conducting this trial. SK, SJ, AFD and RW are employees of Novartis, and RS is a contractor whose services are funded by Novartis.

## Authors' contributions

SH was the principal investigator of the study and contributed to the acquisition of study data and approved the interpretation of data and the study report. Novartis (represented by SK, RS, SJ, AFD and RW) was responsible for the conception and design of the study, and analysis and interpretation of data. All authors had access to the study data, revised the submitted article critically for important intellectual content, and had the responsibility of final approval before submission for publication.

## Pre-publication history

The pre-publication history for this paper can be accessed here:

http://www.biomedcentral.com/1471-2466/11/31/prepub
